# Mixed Method Evaluation of a Graduate Student Teaching and Learning Internship Program

**DOI:** 10.3389/fpubh.2021.762863

**Published:** 2021-11-11

**Authors:** Meredith Tavener, Tazeen Majeed, Tanmay Bagade, Natasha Weaver, Penny Reeves, Shefi Dsilva, Erica L. James

**Affiliations:** ^1^School of Medicine and Public Health, Faculty of Health and Medicine, University of Newcastle, Newcastle, NSW, Australia; ^2^Public Health, Hunter Medical Research Institute, University of Newcastle, Newcastle, NSW, Australia

**Keywords:** graduate student, teaching, learning, mentor, internship, pedagogy

## Abstract

In recognition of the need to better prepare doctoral candidates with teaching and learning competencies, we devised an innovative internship program in the form of a structured apprenticeship and trialed it in public health higher education. The paid internship was comprised of: (i) Mentoring from an experienced educator, (ii) Structured program of education in pedagogy and curriculum design, and (iii) Opportunities for applied experience. Eleven interns completed the apprenticeship in its first 2 years. The mixed method evaluation assessed the impact of the internship on knowledge, skills, and confidence of interns throughout the internship, and included a cost-consequence analysis. Data collection included surveys and face-to-face interviews with interns and mentors. Changes in intern knowledge and skills were analyzed by intern self-ratings pre- and post-internship on 11 performance descriptors. All interns indicated improvement in at least one area of teaching. Interviews indicated general satisfaction, however raised incompatibilities between the unstructured nature of mentoring and intern expectations and preferences. The economic analysis calculated a cost-offset associated with intern-delivered teaching activities of $58,820 (AUD, 2019). The total cost of the program was calculated to be $70,561 (comprising mentor investment AUD$20,436, intern investment AUD$15,126, scholarship “top-up” payment of $5,000 paid to each of the 7 interns AUD $35,000). This Internship is associated with positive impacts for interns across a range of domains at a net total investment of $11,741.

## Introduction

The primary objective of doctoral training programs is the development of researchers; developing the craft of teaching is secondary and something that is hoped will come in due time (1). While the majority of graduating doctoral students seek employment in colleges and universities (2), most future faculty will not find positions at research institutions since only around 6.4 percent of US universities and colleges are considered research institutions (3). Thus, it is clear that most new faculty members will be involved in teaching to some extent (4) and it is therefore reasonable that a doctoral student have some formal training in pedagogy (5). However, despite evidence that providing training in teaching for higher education teachers enhances the teaching experience and boosts self-confidence (6), many university doctoral programs still do not emphasize the importance of preparing doctoral students to teach (7).

There are, however, some initiatives to train doctoral students to teach in academia such as the Preparing Future Faculty initiative (PPF) by the Council of Graduate Schools, USA (8). Under the PPF initiative, North Carolina State University provided teaching and research mentorships, placements, and professional development seminars to sociology doctoral students (9). The evaluation team reported that the program successfully prepared students for an academic role (teaching and research) at Universities (9). A similar program at the University of Maryland's School of Public Health conducted a series of seminars on teaching for doctoral students and noted that 90% of their alumni felt empowered and confident to teach in academia (10). Research from Nigeria reported that higher education teachers with pedagogical training, in addition to their PhD, possessed greater knowledge and pedagogical competencies than teachers without the training (11). Some universities provide similar structured or unstructured training programs for prospective doctoral students. However, the programs may vary across departments, disciplines, and colleges, with little published research on methodology, cost-effectiveness, and the details of the formal pedagogy training programs for doctoral students.

The Educated Citizen and Public Health Initiative, a collaboration of arts and sciences and public health organizations organized in 2006, aims to integrate public health perspectives within a comprehensive liberal education framework and to foster interdisciplinary and inter-professional collaboration (12) meaning that future public health Faculty will likely be required to teach students from a range of disciplines (13). The breadth of practice required by the public health workforce (14) requires higher education programs that can prepare graduates with both theoretical knowledge and practical skills, requiring an understanding of experiential learning opportunities and authentic assessment by Faculty.

Therefore, the current paper describes the evaluation of a model pedagogy-preparation program in public health education run at the University of Newcastle, NSW, Australia.

## Method

### Aims

The aims of this mixed method evaluation were to

1. Assess self-reported changes in intern knowledge, skills and confidence,
2. Calculate the net budget impact of the internship program from an institutional perspective.

### Design

This evaluation used a mixed method, pre-post assessment of intern and mentor experiences, including a cost consequence analysis (15).

### Sample and Recruitment

The internship program commenced in 2018, launched as an opportunity for PhD candidates to differentiate themselves and potentially increase their employability. There were over 350 PhD Candidates enrolled in the School of Medicine and Public Health during the timeframe of the internship. Australian PhD programs focus solely on research training and do not include training in teaching and learning. The internship was advertised to all fulltime candidates and recruited via a competitive process.

Mentors were selected via a call for expressions of interest and were matched with interns on the basis of teaching content. All mentors were experienced educators active in Scholarship of Teaching and Learning.

The interns worked alongside their mentors developing teaching activities and assessment items. They gained real-world experience facilitating tutorials (face to face), facilitating online discussions, and marking assessments, all under the supervision and guidance of mentors. Mentors and interns debriefed following teaching sessions. Where appropriate, interns observed their mentor facilitating a face-to-face tutorial prior to facilitating their own tutorial. Online mentoring and supervision also occurred via the learning management system.

There were 11 interns and 9 mentors involved in the program across 2018 and 2019, from which there were seven interns and six mentors who agreed to be involved and provided data for the evaluation. Ethics requirements for the study meant that individuals who declined to take part did not have to provide reasons as to why.

### Development of the Internship Components

The Internship program design was informed by a review of the literature. In the United Kingdom (UK) and North America the “Teaching Assistant” model is common. Across Australasia, the entry level teaching role is referred to as a tutor. Teaching assistants and tutors obtain practical experience rather than a structured program on how to teach. Korpan et al. (16) developed graduate teaching competencies across three domains: knowledge, skills and social. The Korpan model describes a continual process of reflection and development for beginning educators. The cycle starts with the intern reflecting on the values and goals of the discipline, their previous teaching experience and skills, and successful teaching strategy experiences. They are then encouraged to seek knowledge on both course content, and pedagogical and teaching strategies, as they develop their personal teacher identity. They are then well-placed to develop skills performing teaching-related tasks and managing challenges, and finally, demonstrate the ability to behave professionally and practice effective interprofessional communication.

Based on the Korpan Model, the newly developed internship program was designed as a structured apprenticeship that comprised:

1. Mentoring from an experienced educator who helped the interns analyze their existing teaching experience, compare this to relevant sample position descriptions, identify gaps, and develop a personalized plan;
2. Facilitating access to structured courses on teaching and learning to address the gaps, for example, a short course on writing learning objectives;
3. Practical experience designing assessments and marking rubrics, delivering lectures and tutorials, facilitating online activities and online discussions, and marking assessments.

### Data Collection

Evaluation data were collected via surveys and interviews. Two complementary surveys were developed, one each for interns and mentors, informed by several existing tools (17, 18), and encompassing issues of time commitment, performance and knowledge, communication and social aspects. Also 11 items for both mentors and interns to evaluate intern performance, pre- and post-internship, rated on a 5-point Likert-type scale according to how often the student demonstrated that ability: 1 (Never), 2 (Seldom), 3 (Sometimes), 4 (Usually), 5 (Always) or Not Applicable.

Data for the cost analysis were sourced from the same surveys.

The interview guide was framed by Korpan's teaching assistant competencies. The semi-structured interview format explored internship elements such as intern knowledge, skills and confidence, importance of different internship program components, benefits and challenges, expectations, personal goals and relationships with others. Some interview items were targeted toward mentors, and others toward the interns (Supplementary Material A).

### Ethical Issues

Ethical approval was granted from the University of Newcastle Human Research Ethics Committee at the start of the study before participant recruitment. All participants (interns and mentors) provided written informed consent and where relevant, consented explicitly to the interviews being audio recorded. The invitation to participate in the evaluation was carried out by administrative staff independent to the academic staff involved in the internship program. Completed surveys were returned to administrative staff to anonymise. Participants consenting to interviews, responded directly to author MT who was invited to collect and analyse qualitative data, and who had not been involved in the internship program development or recruitment.

### Analysis

Due to the small number of participants the analysis of survey data was limited to comparing pre- and post-internship rating scores for individual interns. The survey data were also used for the identification and measurement of resource use to inform the economic analysis. Costs of the program were identified as the cost of the scholarship top up payment (AUD$5,000 for each of the 7 interns) and the cost of participants' time to engage with the program. Participants' time was identified and measured via the survey data. This labor time was valued using the human capital approach (19).

The economic benefit of the program was identified as mentors' time freed up as a result of the interns' teaching. The benefits of the program were measured as valuation of the teaching time undertaken by interns, using the opportunity cost method (20). This benefit was calculated as the difference between the cost of the teaching time valued using an average mentor wage and the opportunity cost of the teaching time conducted by interns valued using post graduate scholarship wages as a proxy market value. Return on investment was reported by calculating stakeholder investment (higher education institution, mentors) in the program and evaluating whether the benefits (teaching activities undertaken by interns) exceeded the investment (payments to interns, salary costs of time spent by mentors).

A “coding reliability thematic analysis” was applied to the interview data. The primary steps in this approach were: data collected and coded qualitatively, then verified by members of the research team and illustrated by participant's statements (21, 22).

## Results

### Survey With Interns

Intern surveys reported here are based on responses from seven of the eleven interns (63% participation rate). We compared pre- and post-internship ratings for the five interns who provided sufficient data. Each responding intern rated themselves higher on at least two performance descriptors post-internship. Some interns assessed themselves as having an improvement of 1-point on more than half of the applicable descriptors (2018/2, 2018/3, 2019/1) whereas other interns felt they had a more marked improvement of 2-points in fewer areas (2019/2 in two descriptors related to assessment and 2019/3 in two descriptors related to teaching identity/philosophy). This may reflect differences in tasks chosen by interns or assigned by mentors that either addressed a broad range of skills or a narrower focus on particular teaching activities. A full summary of pre- and post-change is in Table 1.

**Table 1:** Pre- and post-change in intern performance descriptors for each individual intern identified by year.

**Year/Student**	**2018/1**	**2018/2**		**2018/3**		**2018/4**	**2019/1**		**2019/2**		**2019/3**	
**Performance descriptor**	**Post (only)**	**Pre**	**Post**	**Pre**	**Post**	**Post (only)**	**Pre**	**Post**	**Pre**	**Post**	**Pre**	**Post**
Seeks out information about how to prepare lesson plans and executes it		4	5	4	5	2	3	4	4	4		
Seeks out information about how to prepare assessment and executes it	4	4	5	5		4	3	4	3	5		
Seeks out information about how to prepare rubric and executes it	3					3			2	4		
Effectively uses appropriate classroom and online technologies	3	3	5	4	4	4	3	4				
Uses appropriate skills to work with a diverse student population	4	5	5	4	5	4	3	4	5	5		
Gives clear, concise and stimulating presentations/tutorials/lectures	3	5	5	4	4	4	3	4	5		3	4
Attempts to engage with students via Blackboard and provides feedback	3	3	4			4	3	4	4			
Effectively manages classroom	3	5	5	4	4	3	3	4				
Have developed/Are developing your own teaching identity	3	4	5	4	5	4	3	4	4	4	3	5
Seeks out and uses appropriate T & L pedagogies and teaching philosophy	2	4	5	3	4	3	3	3	4	4	3	5
Is critically self-reflective about strategies employed in teaching	2	3	4	3	4	3	4	4	4	4	4	5

*Shading indicates increased rating by 1 point (light) or 2 points (dark).

Only one intern gave both pre- and post- ratings for preparing and executing a rubric, indicating that many PhD students may not have experienced this skill. There were also fewer ratings for classroom-related descriptors (e.g., use of classroom technology and classroom management) possibly due to fewer opportunities for face-to-face teaching (as much of the postgraduate public health curricula is delivered online). Almost none of the pre-internship descriptors had ratings that were <3, possibly indicating reluctance to use the lower end of the scale, and many were rated at the maximum 5. There were no negative pre-post changes.

### Interview Findings

Four interns and three mentors were available and consented for interview, from the 2018 cohort (when interviews were conducted). Interviews with interns and mentors were guided by questions from Supplementary Material A, however, not all questions resonated with participants, and some were not answered. Following anonymisation of data, participant discussions were represented in narrative style to capture the most salient issues discussed but were not “mapped” precisely against the interview guide.

The competency elements most discussed were “abilities” and “skills”, though interns admitted they “*didn't have any*” specific goals in mind as to how to improve either when they began the program, nor clear expectations of what to expect overall. One intern said “*I didn't really have any expectations to be honest… I went into it thinking whatever I get out of it is fantastic*” and others “*thought it would be teaching*.” Interns who had previously gained teaching experience outside of Australia were hopeful for “*a new way of teaching… different culture, different way of doing things*” to broaden their repertoire as educators. There were improvements to confidence: interns who provided lectures were able to showcase their own expertise, and then spoke of receiving praise from their mentor, such as: “*I have changed a lot over this year… I have learned a lot… I feel confident I can be a very good teacher*.”

At the start of the program mentors were required to meet their intern and discuss the course(s) with which they would be assisting. Mentors tried “*not to overwhelm*” the interns, while providing a range of key teaching experiences. One mentor focused on involving an intern in “*development of course content… and face-to-face teaching*” also saying they urged their interns toward “*recording audio feedback*” for students even though it meant some discomfort “…*but we do it anyway, because it's such a time saver and we want (ed) them to have experience in all the key areas of a course*.” One early career mentor described gaining confidence from the internship themself, having never “*had a person who I was giving tasks to, responsible for deciding what they do… checking in to make sure it was done…*.” As a result of their first mentoring experience, the same educator said they “*might change the way we mentor… just modifying it a little bit to suit each individual intern experience… what they need to grow*.”

For a successful internship experience, interns said they should be “…*flexible, goal-oriented and open to constructive criticism and open to learning opportunities, to learning suggestions*.” They expected their mentor to be someone who “*understands the intern's needs… paying attention and being responsive*.” Styles differed between mentors, as did their contact with interns, with one intern feeling that the early phases of their experience required more structure. A reflective journal had been suggested as “*some sort of template to jot down things*” as a record of teaching tasks, and also as “*some measure of pre-and post”* for the intern.

Some of the more challenging aspects of the program, from the intern's perspectives included “*managing the time allocated to teaching, if some periods were busier than others*,” and in general finding that teaching could be “*a lot more time intensive than [they] had anticipated*.” Providing a balanced teaching experience was essential, to avoid “*marking exhaustion*.” One intern confided that “*you just wish the marking was over…that's just part and parcel of teaching*.” Certification courses were also offered as part of the internship, which upskilled interns in key teaching proficiencies such as providing constructive feedback. They proved both popular and challenging, with one intern saying, “*I had to rewire everything I had to learn, for teaching… so many things have changed over the years and learning those new things took a bit of time*.”

Going forward there was support for the internship program. The mentors saw value in the program to “*help develop that pipeline*” in terms of job-ready PhD candidates who have experience and confidence in teaching and learning. Interns suggested a more structured “*feedback procedure*” including more regular meetings with mentors. But also commented that “*every PhD student should have the opportunity to teach somehow*” and “*everyone would benefit from being an intern*.”

### Economic Analysis

Table 2 presents the breakdown of investment and valued economic benefit associated with the internship program. The total cost of the investment by mentors was calculated to be AUD$20,436. The total cost of the investment by interns was estimated to be AUD$15,126 incorporating AUD$13,198 of teaching hours delivered by the interns. A sum of $35,000 was awarded as top-up scholarship payment. The total cost of the investment was calculated to be AUD$70,561. Net costs avoided were calculated as the difference in the total cost of investment and cost-offset realized as a result of interns replacing some teaching activities of mentors. The cost-offset was calculated to be AUD$58,820 over the 2-year period (2018–2019).

**Table 2:** Costs and valued outcomes.

**Economic cost**	**Value**
**Cost of mentor-intern engagement**	
Mentor	$4,543
Intern	+ $1,927
**Cost of mentor-intern teaching activities**	
Mentor	+ $15,893
Intern	+ $13,198
Cost of scholarship top up payments	+ $35,000
Sub-total cost	= $70,561
**Valued benefit**	
Cost offset from avoided teaching-related activities	–$58,820
Final net cost	= $11,741 ($2,574–$25,348)^*^

* *Figures in parentheses represent the potential net cost range from sensitivity analyses using varied wage rates for intern teaching*.

Sensitivity analysis, conducted by varying the wage rate used to value the opportunity cost of the teaching time performed by interns, generated a range in the net cost of the program between $2,574 and $25,348 AUD (2019). Additional economic consequences arising from the program in the short term (and unable to be quantified) included the capacity building of both interns and mentors. Medium term consequences included expected earnings uplift for participating interns.

## Discussion

This study is a first of its kind that analyzed multiple components of a teaching and learning internship program, including its cost-impact. Previous studies assessed the impact of an internship program for PhD students only from a career decision marking point of view (23) or in terms of confidence (6). Most students indicated increased self-assessed skills in developing assessments and developing their identity as educators.

While generally very positive, qualitative data pointed toward the importance of communication between interns and mentors. A more structured approach to feedback was a common discussion thread by interns, perhaps reflective of the intern's feelings of vulnerability given their new teaching and learning role (24). Each intern expressed a strong passion for learning, which may have required greater emotional engagement on the part of the mentor (25). Moreover, even though interns seemed most conscious of developing their abilities and skills as educators, the affective bond that could have developed between intern and mentor should not be overlooked (26).

Being accepted into the internship program held status, with interns proudly including this on their education and academic webpages. The competitive application process for the internship resulted in high achievers being selected—this left them looking for reassurance and a system of measurement for their performance, whereas the Internship program took a less structured, personalized approach to interactions and “assessment.” The selection of academically successful interns may have also led to them being reluctant to rate themselves in the lower half of the rating scale, limiting the capacity to measure improvement.

In addition to seeking more concrete expectations on which to assess their achievements throughout the internship, the interns also sought more opportunities to interact with each other. Increasing opportunities for formal and informal interactions and encouraging the development of a community of practice will be prioritized moving forward. We have also sought to align the internship activities more formally with Fellowship requirements with the Higher Education Academy.

Inclusion of the cost impact analysis provides reassurance to higher education managers that internship programs such as this can be justified from a managerial perspective.

There were limitations to this study. This evaluation was limited to responses from a small number of interns at one school within one academic institution. At this stage we have not conducted any follow-up assessment to evaluate if the interns maintained or improved the knowledge and skills acquired during the program and if they had the opportunity to apply these skills/knowledges in another setting. There is scope to assess employer views on whether participation in the internship increases perceived or actual employability. Our eligibility criteria required the interns to be enrolled as full time PhD candidates who were receiving a scholarship stipend for their living experience, this may have excluded some students from applying and limits generalisability.

A strength of this internship program design is the evidence informed selection of components and the ability for components to be personally tailored to each interns needs. A strength of the mixed methods evaluation was the inclusion of structured reflection from interns, that strengthened our ability to assess the extent of improvement in any personal or professional skills after participating in the internship program.

As only few such programs exist in Australia, this program and the results support the positive benefits for students on both a personal and professional level and at the institutional level and contribute to the body of research on graduate student teaching and learning internship programs in Australian higher education sector by identifying and emphasizing the value of a systematic internship component during their academic career.

Our internship program was a three-way partnership between the interns, the educators and the School of Medicine and Public Health (University of Newcastle) designed to provide an experiential learning opportunity. While these internships provided PhD students with practical and hands-on teaching and learning experiences, introducing them to teaching collaborations within our school, to work with and strengthen ties with experienced educators, continued assessment of this program is imperative. Therefore, there is a need to develop and implement systematic ways to assess the viability, uptake and effectiveness of these initiatives, especially taking into account the valuable insight provided by the students' perceptions of the internship and the role that both the educator and the school played in facilitating their learning. Secondly, the results indicate that there is a need for increased structure. Therefore, our future project and research will focus more on creating effective, structured and engaged professional and graduate school programs and initiatives with a focus on longer term evaluation.

In conclusion, the teaching and learning internship was associated with positive impacts for interns across a range of domains. Other schools of public health should consider offering a similar program to graduate students.

## Data Availability Statement

The datasets presented in this article are not readily available because specific informed consent, and ethics committee approval, would need to be sought and confirmed. Requests to access the datasets should be directed to Meredith Tavener, meredith.tavener@newcastle.edu.au.

## Ethics Statement

The studies involving human participants were reviewed and approved by Human Research Ethics Committee, University of Newcastle. The participants provided their written informed consent to participate in this study.

## Author Contributions

EJ and TM led the conception and design of the study. EJ oversaw the manuscript completion. MT was responsible for the qualitative data collection and analysis and interpretation. PR and SD carried out the economic analysis. NW was responsible for checking and analyzing the survey data. TB provided literature review input and wrote sections of the discussion. All authors contributed to the article and approved the submitted version.

## Funding

The author(s) disclosed receipt of the following financial support for the research, authorship, and/or publication of this article: The internship program was supported by the School of Medicine and Public Health, University of Newcastle, NSW, Australia. The evaluation activities were funded by a grant from the Faculty of Health and Medicine Research Committee.

## Conflict of Interest

The authors declare that the research was conducted in the absence of any commercial or financial relationships that could be construed as a potential conflict of interest.

## Publisher's Note

All claims expressed in this article are solely those of the authors and do not necessarily represent those of their affiliated organizations, or those of the publisher, the editors and the reviewers. Any product that may be evaluated in this article, or claim that may be made by its manufacturer, is not guaranteed or endorsed by the publisher.
