# Supplementary material for: Mixed Method Evaluation of a Graduate Student Teaching and Learning Internship Program
Source: Front Public Health. 2021 Nov 11;9:762863. doi: 10.3389/fpubh.2021.762863 (PMC8631995; doi:10.3389/fpubh.2021.762863)
Supplement: Supplementary file 1 [file Table_1.DOCX]

Supplementary Material A: Semi-structured interview guide

|  | **Key competencies (Korpan et al., 2015)** | | | | |
| --- | --- | --- | --- | --- | --- |
|  |  | **Knowledge** | **Skills** | **Social** | **Abilities** |
| **Original funding application aims / objectives** | Changes in knowledge | Do you feel more critically aware and self-reflective about your work as a result of the program? Why/why not – and what does it mean for your work from now on? (ask interns) | What did you think you would learn? (ask interns)  In what ways do you approach solving problems now? (ask interns)  In what ways did you used to solve problems? (ask interns)  What changes did you see in (name’s) knowledge / skills as a result of the program? (ask supervisor and mentor) | In what ways has the program influenced your interactions with supervisors – mentors – other students/peers? (ask interns) | What personal goals did you set for yourself at the start of the program (ask interns)  What personal goals do you set for yourself now? (ask interns)  What are your current areas of strength? What about before the program – what strengths have changed, and how? (ask interns) |
|  | Changes in skills |  |  |  |  |
|  | Changes in confidence |  |  |  |  |
|  | Relevance and importance of aspects of intern experience | What did you think were the most important and challenging aspects of the program (ask everyone) | What types of experiences should the program include to make interns more competitive and competent? (ask everyone)  What qualities make a good intern? How did/ could the program assist with that? (ask everyone) | What questions did you ask your mentors along the way? (ask interns)  In what ways did your relationship with your supervisor change during the program? (ask interns) | What type of tasks did the *other* interns do, compared to you? (insight into contact with other interns, did they feel isolated, or part of something?) (ask interns)  Did you expect to be doing certain tasks during the program? (ask interns)  What tasks *were* you asked to do as part of the program? (ask interns) |
|  | Experiences of internship for supervisor, mentor and intern | What type of preparation or learning did you do before the program – what was provided to you? (ask everyone)  Describe a negative or disappointing experience you had from the program (interns and mentors)  Describe an experience from the program that you felt was significant (interns and mentors)  What do you feel you’ve learnt from being involved in the intern program? (ask everyone)  How do you know if you did a good job during your internship? What types of feedback did you receive along the way, and from whom? (ask interns) | | | |
|  | Benefits of internship | In what ways was the program relevant to your work? (ask everyone)  From your participation in the program, what types of gains do you feel you’ve made? (ask interns)  What do you think the program has prepared you for? (ask interns) | | | |
|  | Barriers towards successful participation / implementation | What would stand in the way of the program continuing? (ask everyone)  From your experience, what do you feel was not done well in the program – what steps would you take to fix that? (ask interns and mentors) | | | |
